# Abundant PD-L1 expression in Epstein-Barr Virus-infected gastric cancers

**DOI:** 10.18632/oncotarget.9076

**Published:** 2016-04-28

**Authors:** Sarah Derks, Xiaoyun Liao, Anna M. Chiaravalli, Xinsen Xu, M. Constanza Camargo, Enrico Solcia, Fausto Sessa, Tania Fleitas, Gordon J. Freeman, Scott J. Rodig, Charles S. Rabkin, Adam J. Bass

**Affiliations:** ^1^ Department of Medical Oncology, Dana-Farber Cancer Institute, Boston, Massachusetts, USA; ^2^ Department of Medical Oncology, VU University Medical Center, Amsterdam, The Netherlands; ^3^ The Center for Immuno-Oncology, Dana-Farber Cancer Institute, Boston, Massachusetts, USA; ^4^ Department of Pathology, Ospedale di Circolo, Varese, Italy; ^5^ Division of Cancer Epidemiology and Genetics, National Cancer Institute, Rockville, Maryland, USA; ^6^ Department of Molecular Medicine, University of Pavia and Policlinico S. Matteo, Pavia, Italy; ^7^ Department of Medical Oncology, Hospital Clínico Universitario de Valencia, Valencia, Spain; ^8^ Department of Pathology, Brigham and Women's Hospital, Boston, Massachusetts, USA; ^9^ Cancer Program, The Broad Institute of MIT and Harvard, Cambridge, Massachusetts, USA

**Keywords:** EBV-infected gastric cancers, MSI gastric cancer, PD-L1, PD-1 inhibitors

## Abstract

Gastric cancer (GC) is a deadly disease with limited treatment options. Recent studies with PD-1 inhibition have shown promising results in GC, but key questions remain regarding which GC subclass may respond best. In other cancers, expression of the PD-1 ligand PD-L1 has been shown to identify cancers with greater likelihood of response to PD-1 blockade. We here show with immunohistochemistry that Epstein-Barr Virus (EBV)+ GCs (*n* = 32) have robust PD-L1 expression not seen in other GCs. In EBV+ GC, we observed PD-L1 staining in tumor cells in 50% (16/32) and immune cells in 94% (30/32) of cases. Among EBV-negative GCs, PD-L1 expression within tumors cells was observed only in cases with microsatellite instability (MSI), although 35% of EBV-/MSS GCs possessed PD-L1 expression of inflammatory cells. Moreover, distinct classes of GC showed different patterns of PD-L1+ immune cell infiltrations. In both EBV+ and MSI tumors, PD-L1+ inflammatory cells were observed to infiltrate the tumor. By contrast, such cells remained at the tumor border of EBV-/MSS GCs. Consistent with these findings, we utilized gene expression profiling of GCs from The Cancer Genome Atlas study to demonstrate that an interferon-γ driven gene signature, an additional proposed marker of sensitivity to PD-1 therapy, were enriched in EBV+ and MSI GC. These data suggest that patients with EBV+ and MSI GC may have greater likelihood of response to PD-1 blockade and that EBV and MSI status should be evaluated as variables in clinical trials of these emerging inhibitors.

## INTRODUCTION

Gastric cancer (GC) is a deadly disease that, despite its molecular heterogeneity, has been largely treated through uniform approaches. As new profiling approaches and molecular characterization efforts enable better sub-classification of GC, one hope is that these subclasses may help guide optimal selection of therapy, both cytotoxic and biologic therapies. Among biologic agents, there is great enthusiasm regarding use of novel immune checkpoint inhibitors in cancer therapy, most notably those targeting the programmed cell death protein 1 (PD-1) pathway. Together with its ligands PD-L1 and PD-L2, PD-1 participates in regulating the balance between T cell activation and tolerance in response to antigenic stimulation [1]. Indeed, early clinical studies in GC using therapeutic antibodies targeting PD-1 have demonstrated promising results, with response rates of 22–27% of PD-L1-expressing GCs and 12% of PD-L1 negative GCs [2, 3]. However, as only a portion of patients respond to PD-1 inhibition, key questions remain regarding both possible biomarkers that could guide the use of PD-1 inhibitors as well as the potential that distinct biologic classes of GC may differ in sensitivity to these checkpoint inhibitors.

Through a recent comprehensive molecular analysis of 295 gastric adenocarcinomas as part of The Cancer Genome Atlas (TCGA) [4], we and others developed a novel classification system dividing GC into four molecular groups: 1) Epstein-Barr Virus (EBV) positive GC, 2) Microsatellite Instability (MSI), 3) Chromosomal Instability (CIN) or 4) Genomically stable (GS) tumors. Within the context of these groups, the MSI cohort may be predicted to have higher likelihood of response to PD-1 therapies given the responses observed in MSI colorectal cancers [5]. However, in the TCGA study we also identified features of the EBV+ cancers that suggested the potential for the PD-1 pathway to be relevant. Specifically, we observed that 15% EBV+ GCs possessed genomic amplification of chromosomal region 9p24.1, the locus of genes encoding PD-1 ligands PD-L1 and PD-L2. As PD-1 blockade has been found to be most effective in PD-L1+ tumors [3, 6–8] these results suggest that specifically EBV+ GC may be prime candidates for PD-1 directed therapy.

EBV is a human herpes virus that contributes to the development of a diversity of malignancies such as B and T cell lymphomas, nasopharyngeal carcinomas and ~9% of gastric adenocarcinomas [9]. Although the precise role of EBV in the carcinogenic process is not fully understood, EBV+ GCs form a distinct clinicopathologic subgroup with a longer survival [10], results ascribed to a prominent CD8+ cytotoxic T-cell infiltrate as part of an EBV-directed immune response [11]. This predilection to enhanced survival and a strong inflammatory infiltrate is reminiscent of findings of MSI colorectal carcinomas. In the setting of this strong inflammatory response, activation of pathways such as PD-1 to attenuate the immune response may be particularly essential for EBV+ disease. Enhanced expression of PD-L1 and PD-L2 by 9p24.1 amplification can therefore be a powerful tool of EBV+ GCs to evade immune attack.

Besides amplification of 9p24.1, additional features of EBV+ gastric cancers may possess alternative mechanisms able to induce PD-L1 expression. EBV positive classical Hodgkin's lymphoma, for instance, have increased PD-L1 expression levels as a result of EBV induced AP-1 expression and JAK/STAT signaling [12]. Additionally, IFN-γ released by tumor infiltrating T cells can directly induce PD-L1 expression in tumor and immune cells [13–15]. As IFN-γ plays an important role in the innate and adaptive immune responses in the defense against EBV infection [16], an IFN-γ response may well be elicited by EBV-infection in GC. Interestingly, preliminary results from retrospective studies have shown that besides PD-L1 expression a pre-existing interferon-mediated adaptive immune response is associated with response to PD-1 blockade [17–19]. Together, these data lead us to hypothesize that EBV may mark a group of GCs with greater likelihood of benefit from anti-PD-1 directed therapy.

In this study, we investigate the status of candidate markers to guide the use of PD-1 therapy, immunohistochemical analysis of PD-L1 status and gene expression based analysis of IFN-γ immune signatures, in GCs with and without EBV-positivity. The primary question we hope to address is if markers that suggest greater likelihood of potential response to PD-1 blockade are a broad feature of EBV+ GCs or are more restricted to the smaller subset of EBV+ tumors with 9p24.1 amplification.

## RESULTS

### EBV+ GCs have abundant PD-L1 expression in tumor and tumor infiltrating immune cells

**Figure 1:**
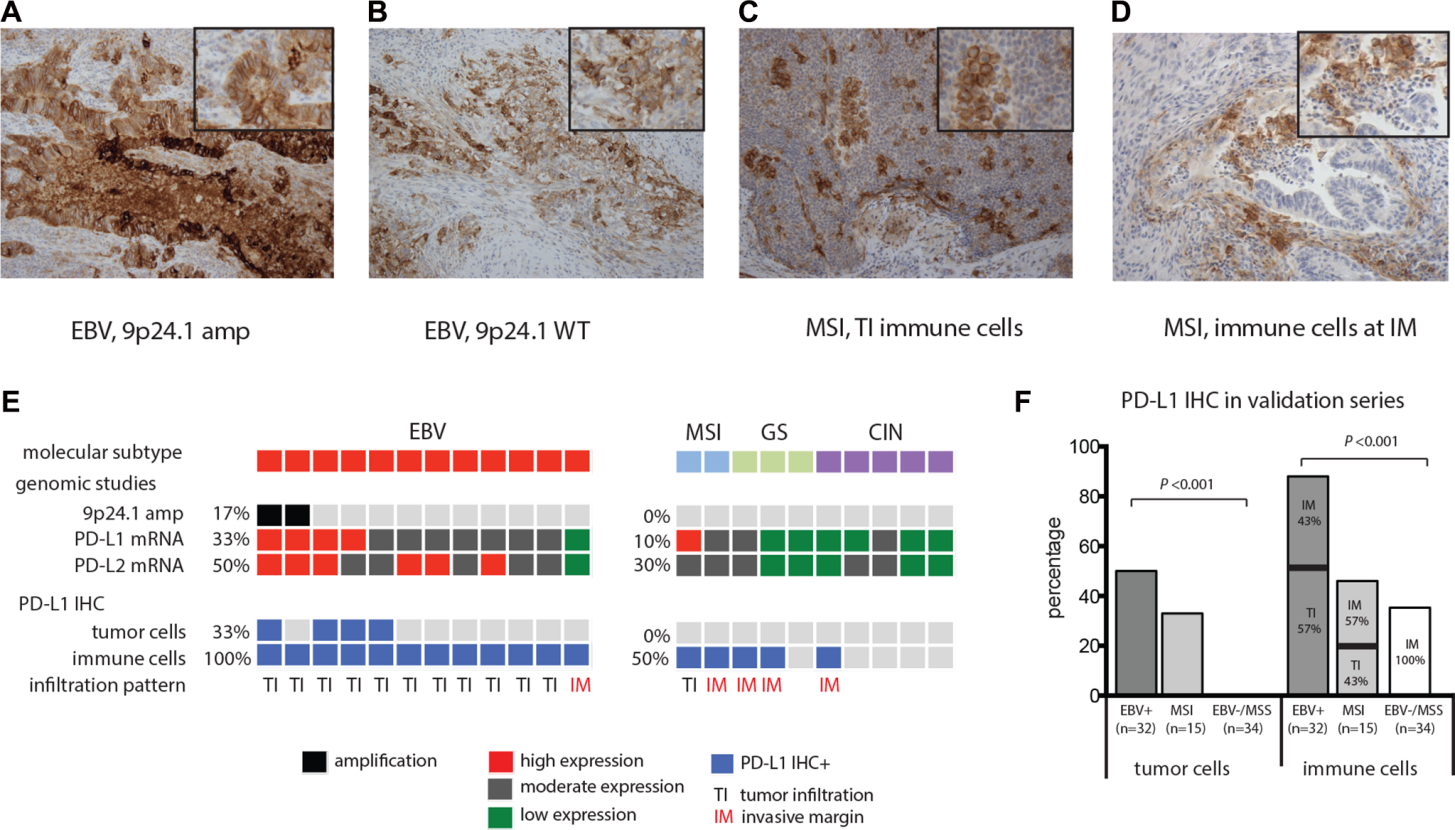
PD-L1 expression (IHC) staining of whole tissues slides of FFPE EBV+ GC and MSI GCs (**A**) EBV+ GC with 9p24.1 amplification (amp) has abundant PD-L1 expression of tumor cells. (**B**) EBV+ GC and MSI GC (**C**) with PD-L1+ immune cells with a tumor infiltrating infiltration pattern (TI). (**D**) MSI GCs with PD-L1+ immune cells exclusively at the invasive margin (IM) (D). Magnification 20×, insert indicates area of higher magnification. Associations between EBV status and PD-L1 expression in TCGA study (**E**) and validation series (**F**).

In order to determine whether PD-L1 expression is restricted to 9p24.1 amplified EBV+ GCs we collected a sample series of 12 EBV+ and 10 EBV negative GCs (2 microsatellite instable (MSI), 3 genomic instable (GS), 5 chromosomal instable (CIN)) from the TCGA study, using cases in which we were able to retrieve tissue slides [4]. In these cases, the EBV status was ascertained by a combination of analysis of mRNA sequencing and DNA sequencing and confirmed by *in situ* hybridization using probes against Epstein-Barr encoded RNA 1 (EBER1) as previously described [1, 16]. We evaluated PD-L1 expression by immunohistochemistry (IHC) (Figure 1A–1D and Table 1). In this series, 2 out of 12 (17%) EBV+ GC had 9p24.1 amplification and both cancers showed increased PD-L1 and PD-L2 mRNA expression (Figure 1E). Potentially due to heterogeneity of PD-L1 protein expression within the tumor [20], only one of these two EBV+ GCs showed PD-L1 expression by IHC. However, also EBV+ GCs without 9p24.1 amplification had PD-L1 positive tumor cells in 3 out of 10 (33%) cases. By contrast, all 10 EBV negative GCs from this small dataset lacked apparent PD-L1 staining of carcinoma cells. Beyond the presence of PD-L1 in tumor cells in the EBV+ cases, all 12 evaluated EBV+ GCs possessed PD-L1+ immune cells, a finding found in only five out of 10 (50%) EBV negative GCs. Beyond the differential presence of PD-L1+ cells, these tumor types showed different patterns of expression. In 11 out of 12 EBV+ GCs, PD-L1+ immune cells were observed to infiltrate the tumor (TI), while in 4/5 PD-L1+/EBV negative GCs, PD-L1+ immune cells stayed at the invasive margin (IM) (Figure 1C–1E).

**Table 1:** Clinical and pathologic characteristics and PD-L1 expression status of EBV+ and EBV− gastric adenocarcinomas

			TCGA series			validation series			
			EBV+, % (*n* = 12)	EBV−, % (*n* = 10)	*P*	EBV+, % (*n* = 32)	EBV− (*n* = 49)		*P*
							MSI, % (*n* = 15)	MSS, % (*n* = 34)	
Sex	male		83	80		72	40	68	< 0.001
Age	years (mean)		65	64		66	73	67	
Stage (AJCC 6th ed.)	I		0	0		47	60	21	
	II		42	60		44	20	39	
	III		50	30		3	20	32	
	IV		8	10		6	0	9	0.015
Anatomical site	GEJ-cardia		25	40		13	0	9	
	fundus-corpus		58	30		59	13	26	
	antrum		17	30		9	87	53	
	pylorus					0	0	0	
	stump					19	0	9	
	unspecified					0	0	3	< 0.001
Lauren classification	intestinal		50	70		81	87	79	
	diffuse		8	20					
	mixed		25	10		19	13	21	< 0.001
PD-L1 expression	tumor cell		33	0		50	33	0	< 0.001
	immune cell		100	50	0.010	94	47	35	< 0.001
		TI	92	20		57	43	0	
		IM	8	80	0.010	43	57	100	0.003

TCGA, The Cancer Genome Atlas; EBV, Epstein-Barr Virus; MSI, microsatellite instable; MSS, microsatellite stable; GEJ, gastro-esophageal junction; AJCC, American Joint Committee on Cancer; TI, tumor infiltration; IM, invasive margin.

As these observations were limited to a small dataset, we next validated PD-L1 expression results in an independent cohort of 81 GCs, 32 EBV+ and 49 EBV negative GCs identified by *in situ* hybridization [21]. Among the 49 EBV-negative cases were 15 microsatellite instable (MSI) GCs (Table 1). Consistent with other sample sets [10, 21, 22], the EBV+ GCs in this collection showed a male predominance (*P* < 0.001), predisposition to a proximal tumor location (*P* < 0.001) and a lower TNM classification (*P* < 0.015) compared to EBV negative GCs. As single only a single tissue slide was available for analysis, we restricted our testing to PD-L1 (as this marker is currently being evaluated as a guide to use of PD-1 therapy in GC). In the EBV+ samples, we identified PD-L1+ tumor cells in 50% (16/32) and PD-L1+ immune cells in 94% (30/32) of tumors. By contrast, EBV negative GCs had significantly less PD-L1 staining in tumor cells (10% (5/49), *P* < 0.001) and immune cells (39% (19/49, *P* < 0.001). When we broke out the EBV negative GC by MSI status we found that MSI GCs had PD-L1+ tumor and immune cells in 33% (5/15) and 46% (7/15) of cases respectively, which was higher than EBV negative microsatellite stable (MSS) GCs that had no PD-L1 positive tumor cells (0% (0/34), *P* < 0.001) and PD-L1 positive immune cells in only 35% of cases (12/34, *P* < 0.001). Again, PD-L1+ immune cells had a tumor infiltrating patterns in 57% of PD-L1+/EBV+ cases and 43% in MSI GCs which was 0% in PD-L1+/EBV- /MSS gastric cancers (*P* < 0.001) (Figure 1F).

### EBV+ and MSI gastric cancers have enrichment of an IFN-γ immune response signature

**Figure 2:**
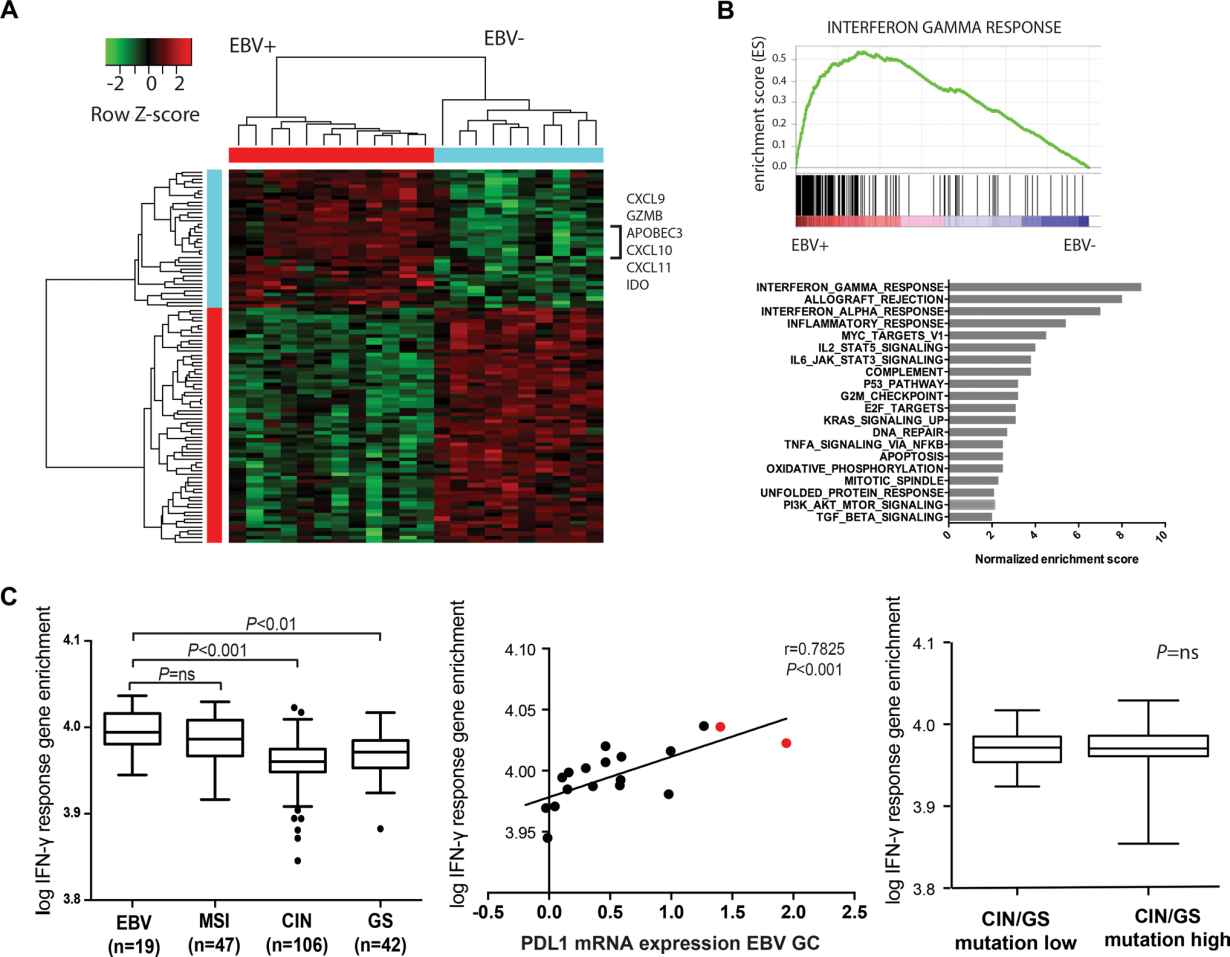
Supervised hierarchical cluster analyses (**A**) and gene set enrichment analyses (GSEA) (**B**) shows enrichment of IFN-γ response genes in EBV+ GCs. (**C**) Single sample gene set enrichment (GSE) analyses shows IFN-γ GSE in EBV+ and MSI GCs (left), IFN-γ GSE in GCs with and without 9p24.1 amplification (middle, cases with 9p24.1 amplification indicated in red) and absence of an association between IFN-γ GSE and mutational load in genomic stable (GS) and chromosomal instable (CIN) GCs (right).

We next determined whether PD-L1 expression in EBV+ GCs was associated with a specific immune signature. Therefore we first studied the transcriptional landscape of the same EBV+ (*n* = 12) and EBV negative GCs (*n* = 10) from the TCGA study. Supervised clustering of genes that discriminate the EBV+ tumors identified IFN-γ signaling genes CXCL9, CXCL10, CXCL11, IDO and GZMB among the top 40 most enriched genes (Figure 2A). A GSEA query for genetic signatures enriched in the EBV+ GCs found an IFN-γ response signature to be the most strongly enriched in the EBV+ group (Figure 2B).

We next sought to look at the strength of the IFN-γ gene signature between different molecular GC subtypes within a bigger series of 214 individual GCs from the TCGA study using single-sample GSEA (ssGSEA). These results demonstrated that besides EBV+ GCs (*n* = 19) MSI GCs (*n* = 47) have high IFN-γ response gene expression compared to GS (*n* = 42) and CIN (*n* = 106) GCs (*P* < 0.001). As expected, IFN-γ GSE was strongly associated with PD-L1 mRNA expression within all GC subgroups (*P* < 0.001). Furthermore, within these subgroups there was no association between IFN-γ signature and total number of mutations (Supplementary Figure 1) as obtained from whole exome sequencing data.

## DISCUSSION

In this study we aimed to evaluate PD-L1 expression and the presence of an interferon-mediated adaptive immune response in distinct gastric cancer subtypes and showed that particularly EBV+ GCs have profound PD-L1+ tumor cells and tumor infiltrating immune cells. PD-L1 expression was not restricted to GCs with 9p24.1 amplification, which indicates that EBV+ cancers have multiple mechanisms to induce PD-L1 expression and suggests that PD-1-driven immune evasion may more broadly play an important role in EBV gastric cancers.

Besides EBV+ GCs, also MSI GCs have PD-L1 expression in tumor and immune cells in 33% and 45% of cases respectively, in contrast to EBV negative microsatellite stable (MSS) GCs that did not have PD-L1+ tumor cells and PD-L1+ immune cells in only 35% of GCs. Remarkably, beyond the differential presence of PD-L1+ cells in EBV+ and EBV negative GCs, we observed a difference in infiltration pattern of PD-L1 positive immune cells; while PD-L1+ immune cells were able to infiltrate the center of EBV+ and MSI GCs, in EBV negative MSS GCs PD-L1+ immune cells stayed mainly at the invasive margin.

Although molecular predictors of clinical benefit from PD-1 inhibitors remain uncertain, PD-1 blockade has been found to be most effective in PD-L1+ tumors or tumors whose PD-L1+ immune cells are able to infiltrate the tumor center rather than remain at the invasive margin [6–8]. Furthermore, retrospective studies have shown that an antecedent interferon-mediated adaptive immune response is associated with response to PD-1 inhibitors in melanoma and head and neck cancer [17–19]. Also a recent study including 33 GCs showed that a tumor immune microenvironment dominated by IFN-γ and T-cell receptor (TCR) signaling was significantly associated with clinical benefit from Pembrolizumab [19].

With mRNA sequencing analyses we here showed that compared to EBV negative GCs, EBV+ GCs indeed have strong enrichment of IFN-γ response genes CXCL9, CXCL10, CXCL11, IDO and GZMB. The combination of PD-L1 positivity and enrichment for an IFN-γ signature in EBV+ GCs suggests the potential for PD-L1 expression and activation of the PD-1 pathway to be a critical mechanism in these tumors to control an antecedent cytotoxic anti-tumor immune response, which increases the likelihood of response to PD-1 blockade in this GC subtype.

Interestingly, besides EBV+ GCs, we also observed that MSI GCs have high IFN-γ response gene expression, perhaps reflecting the large lymphocyte infiltrate that is typical for mismatch-repair deficient cancers with a high mutational load. Whether MSI GC will respond as well to anti-PD-1 directed therapy as MSI colorectal cancers [5] needs further investigation.

Furthermore, although IFN-γ response gene expression occurs less frequent in genomic stable (GS) and chromosomal instable (CIN) GCs, we also noted the presence of PD-L1 expression in inflammatory cells in a significant minority of these tumors. Moreover, PD-L1 mRNA expression was associated with an IFN-γ signature. This finding is in agreement with a recent study of 34 GCs [23] that also showed an association between PD-L1 expression by both tumor cells and immune cells and an increased density of CD8+ T-cells in EBV negative GCs. It is unknown what initiates the interferon-mediated immune response in GS and CIN GCs. Although mutation derived neoantigens are important drivers of tumor immunogenicity and have been associated with clinical response to PD-1 directed therapy in lung cancer and colorectal cancer [5, 24], in our series no association between IFN-γ response gene expression and a high mutational load was observed.

In summary, we here show that PD-L1 expression by tumor or tumor infiltrating immune cells is a general phenomenon in EBV+ GCs and MSI GCs. Enrichment of an IFN-γ gene expression signature indicates a interferon-mediated adaptive immune response in EBV+ and MSI GCs, which provides a strong rationale for testing of PD-1 blockade in this patient population and for evaluating EBV status along with MSI status as key variables in immunotherapy trials in gastric cancer.

## MATERIALS AND METHODS

### Patient series

In order to study PD-L1 protein expression in EBV+ gastric cancers in relation to 9p24.1 amplification, formalin-fixed, paraffin-embedded (FFPE) tissue sections were retrieved from tumor block of 12 EBV+ and 10 EBV negative gastric cancers (2 microsatellite instable (MSI), 3 genomic instable (GS), 5 chromosomal instable (CIN)) that were part of a recent comprehensive molecular analysis of 295 gastric adenocarcinomas as part of The Cancer Genome Atlas (TCGA) [1]. Tissue samples were collected by contacting all Tissue Source Sites (TSS) that had contributed 1 or more cases of EBV+ GC to the TCGA study. Five out of seven TSS agreed to participate in the current study and collected whole tissue slides of all EBV+ cases and an equal number of randomly selected EBV-negative cases. Ultimately, we were able to collect 13 of the requested EBV+ samples and 11 of the EBV-negative samples. Due to absence of tumor tissue on one tissue slide and failure of mRNA sequencing in another sample, 1 EBV+ GCs and 1 EBV negative GCs were excluded for further analyses.

In this series EBV status was determined by whole-genome, whole exome, mRNA and miRNA sequencing and confirmed by *in situ* hybridization using probes against Epstein-Barr encoded RNA 1 (EBER1) as previously described [1, 16]. 9p24.1 amplification status was based on array-based somatic copy number analysis. PD-L1 and PD-L2 mRNA expression status was based on mRNA sequencing and annotated as high expression (>75th percentile), moderate expression (25–75th percentile) and low expression (< 25th percentile).

An independent series of FFPE tissue sections to validate PD-L1 expression results was composed of 32 EBV+ and 49 EBV negative, among which 15/49 EBV negative GCs were microsatellite instable (MSI) [21]. EBV+ cases were selected from a bigger patient series from Italy based on tissue availability. A representative group of EBV negative cases were selected as control. In this series EBV status was determined by *in situ* hybridization for EBER1 and MSI status was assed at Bat 25, Bat 26, BAT40, D5S346 and D2S123 loci [21]. Tumors with instability involving at least two of the five loci were classified as microsatellite instable (MSI). Selection of cases was based on availability of material. Clinical and pathologic characteristics of both series are described in Table 1. All patients were not treated with prior chemotherapy or radiotherapy and provided informed consent. Local Institutional Review Boards approved tissue collection.

### Immunohistochemistry

PD-L1 IHC was performed using mouse anti-human PD-L1 (clone 405.9A11) as previously described [8, 17]. 405.9A11 recognizes an epitope in the PD-L1 cytoplasmic domain and reactivity confirms the expression of full-length PD-L1 protein. The immunohistochemical assay was extensively validated using FFPE cell line controls known to be positive or negative for PD-L1 expression by flow cytometry [18]. PD-L1 staining was observed in membranes and/or cytoplasm of tumor cells and immune cells and was considered positive if ≥ 5% of tumor cells had membranous staining or any positive immune cells with an intensity of 2+ or 3+ (0 = no staining; 1+ weak/equivocal staining; 2+ moderate, definitive staining; 3+ strong, definitive staining) as reported previously [8]. Besides intensity also the tumor infiltration pattern was annotated. TI; PD-L1+ immune cells infiltrate tumor. IM: PD-L1+ immune cells are restricted to the invasive margin. Scoring was performed by an expert immunopathologist (XL) who was blinded for EBV status.

### Gene set enrichment analysis

Differentially expressed genes between EBV+ and EBV negative GCs were identified using TCGA mRNA-sequencing data of 214 GCs of different GC subgroups; 19 EBV+ GCs, 42 MSI GCs, 42 GS GCs and 106 CIN GCs (http://gdac.broadinstitute.org/) [1]. Gene expression analyses were done using the R package “limma”, with adjustment for false discovery rate. Top differentially expressed genes were used for hierarchical clustering using heatmap.2 function available in the “gplots” R package. Enrichment for differential regulated gene sets was computed by Gene Set Enrichment Analysis (GSEA). Hallmark gene sets were downloaded from the MSigDB database (http://software.broadinstitute.org/gsea/msigdb). Enrichment scores of the IFN-γ gene set for each case were obtained by single-sample GSEA using the R code from Genepattern (http://www.broadinstitute.org/cancer/software/genepattern/). Enrichment scores of the IFN-γ gene set were correlated to GC subgroup status, PD-L1 mRNA expression status and total number of mutated genes. The total number of mutated genes were obtained from whole exome sequencing data (http://gdac.broadinstitute.org/) [1].

### Statistical analyses

Associations between clinical, pathological or molecular characteristics were analyzed using two-tailed Student *t*-test and Pearson's chi-2 or Fisher's Exact test where appropriate. Associations between IFN-γ signatures and GC subgroups, PD-L1 mRNA expression status and mutational load were analyzed using Spearman's rank order correlation and one-way Anova. All *p*-values are two sided, and a *p*-value < 0.05 was considered statistically significant.

## SUPPLEMENTARY MATERIAL FIGURE


