# Supplementary material for: Abundant PD-L1 expression in Epstein-Barr Virus-infected gastric cancers
Source: Oncotarget. 2016 Apr 28;7(22):32925–32. doi: 10.18632/oncotarget.9076 (PMC5078063; doi:10.18632/oncotarget.9076)
Supplement: Supplementary file 1 [file oncotarget-07-32925-s001.pdf]

Abundant PD-L1 expression in epstein-barr virus-infected gastric cancers

Supplementary Materials

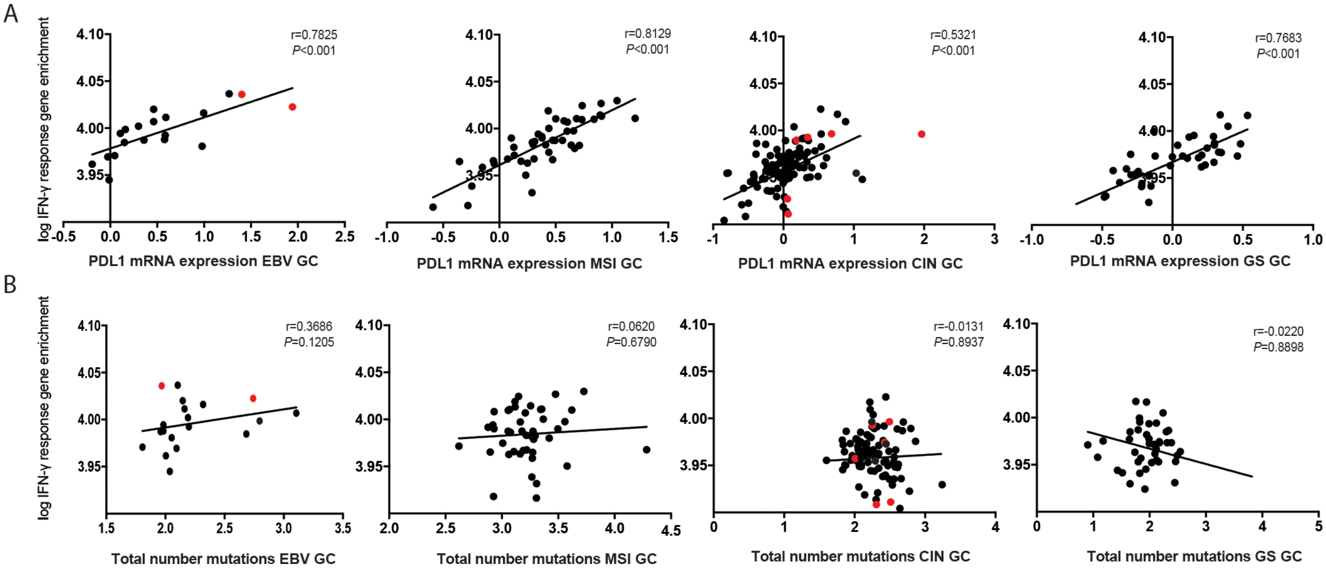

Supplementary Figure S1: Association between IFN-γ GSE and PD-L1 mRNA expression (A) and total number of somatic mutations (B) per gastric cancer subtype. Cases with 9p24.1 amplification are indicated in red.
